# Skin prick testing with extensively heated milk or egg products helps predict the outcome of an oral food challenge: a retrospective analysis

**DOI:** 10.1186/1710-1492-8-5

**Published:** 2012-07-11

**Authors:** Zein Faraj, Harold L Kim

**Affiliations:** 1 Michael G. DeGroote School of Medicine, McMaster University, Hamilton, ON, Canada; 2 Department of Medicine, University of Western Ontario, London, ON, Canada

## Abstract

**Background:**

Cow’s milk and hen’s egg are the most frequently encountered food allergens in the pediatric population. Skin prick testing (SPT) with commercial extracts followed by an oral food challenge (OFC) are routinely performed in the diagnostic investigation of these children. Recent evidence suggests that milk-allergic and/or egg-allergic individuals can often tolerate extensively heated (EH) forms of these foods. This study evaluated the predictive value of a negative SPT with EH milk or egg in determining whether a child would tolerate an OFC to the EH food product.

**Methods:**

Charts from a single allergy clinic were reviewed for any patient with a negative SPT to EH milk or egg, prepared in the form of a muffin. Data collected included age, sex, symptoms of food allergy, co-morbidities and the success of the OFC to the muffin.

**Results:**

Fifty-eight patients had negative SPTs to the EH milk or egg in a muffin and underwent OFC to the appropriate EH food in the outpatient clinic. Fifty-five of these patients tolerated the OFC. The negative predictive value for the SPT with the EH food product was 94.8%.

**Conclusions:**

SPT with EH milk or egg products was predictive of a successful OFC to the same food. Larger prospective studies are required to substantiate these findings.

## Background

Although estimates of prevalence are heterogeneous in medical literature, cow’s milk and hen’s egg are consistently reported as two of the most common food allergens in the pediatric population [1]. The diagnostic investigation for food allergy commences with skin prick testing (SPT) with commercial extracts of suspected allergens. In cases of true IgE-mediated allergic reactions, a localized cutaneous swelling in the form of a ‘wheal’ usually ensues. Typically, negative SPTs are followed by an oral food challenge (OFC), the gold standard, to definitively rule out food allergy.

The standard management of food allergy is strict avoidance of the confirmed allergen [2]. For both milk-allergic and egg-allergic patients, this restriction limits dietary options. Undoubtedly, adherence to this regimen can be burdensome, limits dietary variety and negatively impacts quality of life.

In recent years, evidence has emerged suggesting that the majority of children with milk and/or egg allergy can tolerate these foods when they are extensively heated (EH) [3,4]. Extensive heating alters the allergenic proteins to which IgE antibodies typically form and allergenicity is attenuated in cases of certain allergens such as milk and egg [5]. Furthermore, it has been suggested that exposure may be therapeutic and extended delay in introduction may be detrimental by increasing risk of allergy and delaying the development of tolerance [6]. It has also been proposed that development of tolerance to EH products precedes tolerance to the unheated product by several years [7]. These findings imply that milk-allergic and egg-allergic patients’ diets may have been unnecessarily restricted. Nonetheless, a subset of these children are truly allergic to both EH and non-EH milk and/or eggs, and will react to both forms in oral challenges. This may be due to the presence of heat-stable proteins that can maintain their allergenicity despite extensive heating [5]. It is important to consider whether the advantage of potentially being able to consume EH products is worth the risk taken during the OFC. For example, one study reported that 73% of egg-reactive children who had a positive SPT to commercial extracts were able to tolerate egg baked in a muffin and cooked in a waffle during a physician-supervised OFC [4]. The subjects who did not tolerate the EH egg were at risk of experiencing an anaphylactic reaction.

This study aimed to evaluate whether a negative fresh food SPT with the EH milk or egg products serves as a reliable marker in predicting tolerance to an OFC with the same product in the outpatient clinical setting.

## Methods

A retrospective chart review was performed on all patients undergoing cow’s milk or hen’s egg skin prick testing at a single allergy and immunology clinic in Kitchener, Ontario during a 2 year time period from 2009–2011. Patients were deemed eligible if they were between the ages of 6 months and 18 years at the time of an initial positive SPT to cow’s milk and/or hen’s egg commercial extracts, had a subsequent negative SPT to the EH version of the allergen, and proceeded to an OFC with the EH milk or egg product. An SPT was considered positive if the wheal’s diameter was at least three millimeters larger than the negative control test. All eligible subjects had either previously experienced an allergic reaction to milk or egg or had a positive SPT predictive of an allergy. Subjects with a history of reaction to baked milk or egg products were excluded from the study.

The anterior surface of the forearm was used for skin prick testing with commercially-prepared cow’s milk and hen’s egg extract testing solutions (Omega Labarotories Limited). A drop of egg or milk extract, a negative control, and a positive histamine control were applied to the forearm. The drops were pricked using a Hollister-Stier lancetter and the tests were read after 15 minutes.

Later, SPT was performed with fresh food extracts prepared from an EH milk or egg product. The EH product consistently used was a wheat-based muffin baked with either one third of an egg (both egg yolk & white) per muffin at 350°F for 30 minutes or 40 mL of homogenized milk per muffin at 350°F for 30 minutes. These amounts were deemed greater than would be found in the ingredients of an average baked good of a similar serving size. The muffins were prepared at home by the caregivers and brought to the appointment. Approximately 1 gram of the muffin was thoroughly mixed with 10 ml of water using a tongue depressor. A drop of the slurry was placed on the forearm and pricked with a Hollister-Stier lancetter. The tests were read after 15 minutes.

The OFC was performed in 30-minute intervals, with administration of each dose only if the preceding portion was tolerated. Initially 10% of the muffin was administered for ingestion, followed by three portions of 30%. Subjects were monitored in the clinic for 60 minutes following the OFC.

The SPT and OFC for all subjects were performed under direct supervision of an allergist in his outpatient clinic.

## Results

**Figure 1:**
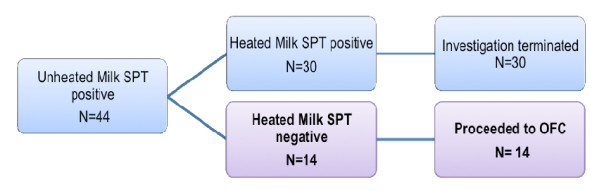
**Milk-allergic subjects meeting the inclusion criteria.***SPT,* Skin Prick Test; *OFC,* Oral Food Challenge. Bold met the inclusion criteria.

**Figure 2:**
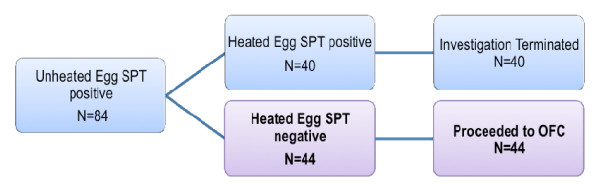
**Egg-allergic subjects meeting the inclusion criteria.***SPT,* Skin Prick Test; *OFC,* Oral Food Challenge. Bold met the inclusion criteria.

Of the 128 subjects found to have a positive SPT to unheated cow’s milk or hen’s egg, 58 subjects (median age 3.5 years; range 1.25–13 years) met the remainder of the inclusion criteria for this study. Fourteen were milk-allergic whereas 40 were egg-allergic (Figures 1 and 2). The median age at first reaction was 1 year (range 0.5–7 years). Initial allergic reaction varied but included cutaneous symptoms such as hives, pruritis or flushing (84.5%), upper airway symptoms including sneezing or throat symptoms (10.3%), lower airway symptoms including wheeze-bronchospasm or respiratory distress (3.5%), gastrointestinal symptoms such as nausea, vomiting, cramping abdominal pain, bloating, diarrhea (22.4%), and cardiovascular symptoms evidenced by dizziness or hypotension (3.5%). Anaphylaxis was reported in 8.6% of patients at initial reaction (Table 1).

**Table 1:** Characteristics, history and symptoms of subjects included in the study, reported both as a summative total and as milk-allergic or egg-allergic independently

**Characteristic**	**All subjects**	**Milk-Allergic only**	**Egg-Allergic only**
Total subjects (%)	58 (100%)	14 (24.1%)	44 (75.9%)
Male sex (%)	34 (59%)	12 (86%)	22 (50%)
Age (y), median (range)	3.5 (1.25–13)	3.5 (2–9)	3.5 (1.25–13)
Co-morbidities (%)			
Asthma	18 (31.0%)	6 (42.9%)	12 (27.3%)
Allergic Rhinitis	17 (29.3%)	4 (28.6%)	13 (29.6%)
Atopic Dermatitis	30 (51.7%)	7 (50%)	23 (52.3%)
Other food allergy	49 (79.3%)	12 (85.7%)	34 (77.3%)
Family history of atopy	43 (74.1%)	11 (78.6%)	32 (72.7%)
Age at first reaction (y), median (range)	1 (0.5–7)	0.5 (0.5–1.25)	1 (0.5–7)
Symptoms at first reaction (%):			
Cutaneous	49 (84.5%)	11 (78.6%)	38 (86.4%)
Upper Respiratory	6 (10.3%)	3 (21.4%)	3 (6.8%)
Lower Respiratory	2 (3.5%)	2 (14.3%)	0 (0%)
Gastrointestinal	13 (22.4%)	5 (35.7%)	8 (18.1%)
Cardiovascular	2 (3.5%)	2 (14.3%)	0 (0%)
Anaphylaxis	5 (8.62%)	4 (28.6%)	1 (2.3%)

Other atopic conditions were highly prevalent in children with cow’s milk and hen’s egg allergies. Co-morbid asthma was found in 31.0% of subjects, allergic rhinitis in 29.3%, and history of current or resolved atopic dermatitis in 51.7%. A positive family history of atopy was reported in 74.1% of the subjects and multiple food allergies reported in 79.3% (Table 1).

**Figure 3:**
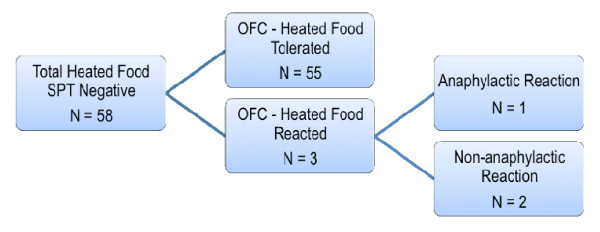
**Summative results of both milk and egg allergic subjects to an OFC, including anaphylactic reactions.***SPT,* Skin Prick Test; *OFC,* Oral Food Challenge.

The median wheal size for initial SPT testing with unheated milk or egg products was 5 mm (range 3 – 9 mm). All the subsequent fresh SPTs with the EH milk or egg products were negative as per the inclusion criteria. This was followed by an OFC with the same food. Fifty-five children (94.8%) did not experience any allergic reaction to EH milk or eggs. Three children (5.2%) reacted to the EH product and were therefore allergic to the food in both forms. All reactions to OFC occurred in subjects from the egg-allergic cohort (Figure 3).

Only one of the three reactions was anaphylactic in nature, as defined by the clinical criteria from the second symposium on anaphylaxis [8]. This 2.25 year old patient experienced eye swelling after 40% of the muffin and was given a dose of diphenhydramine. Fifteen minutes later, symptoms worsened to include vomiting. An hour later, the patient became dyspneic and drowsy. While oxygen saturation level fell to 93% on pulse oximetry, he maintained a pulse of 100–125 and lungs remained clear to auscultation. Epinephrine was administered twice. This was the child’s first exposure to egg and therefore there was no previous history of an allergic reaction to egg. The wheal diameter of his SPT to commercial hen’s egg was mild at 3 mm.

The two non-anaphylactic reactions were in children aged 2.5 and 1.75 respectively. The first of the two non-anaphylactic patients developed hives, followed by pallor and dizziness but maintained stable vital signs after 40% of the muffin. Epinephrine was also administered, and his symptoms resolved within 15 minutes. Previous history of anaphylaxis was documented in this patient, with symptoms of a rash, throat tightening and vomiting. His positive SPT to commercial hen’s egg was recorded at 5 mm. The second patient developed mild peri-oral erythema after consuming one tenth of the muffin, and no medical interventions were necessary; the challenge was terminated and his symptoms resolved. The history of his initial reaction to egg was limited to a mild rash, and his positive SPT to commercial hen’s egg was mild at 3 mm.

## Discussion

The vast majority of subjects with a negative fresh food SPT of the EH food product had successful OFCs to the baked muffins. Fifty-five of the 58 patients tolerated the oral challenge and were encouraged to re-introduce baked eggs or milk into their diets, whereas three reacted and were assumed to be allergic to the food products in all forms. Only one of the three had an anaphylactic reaction (Figure 3). The negative predictive value for the SPT with the extensively heated food product was 94.8%.

This study is the first to propose performing a fresh food SPT in children with milk and egg food allergies in order to predict outcomes of an OFC with EH food. This may serve as a practical marker for children likely to be tolerant of EH milk and egg OFCs. Although previous studies have shown that the majority of food-allergic children tolerate the same foods in their EH forms, the proportion of children experiencing an allergic reaction during the OFC remained significant. Reaction rates of 23% were reported by two separate studies, one challenging milk-allergic children to an OFC with EH milk and the second challenging egg-allergic children to an OFC with EH egg products. Twenty-three out of a sample size of 100 reacted in the milk allergy study, whereas 27 out of 117 reacted in the egg allergy study [3,4]. In our study, the risk of reaction during the diagnostic process was reduced from 23% to 5% by performing an SPT with EH foods prior to their OFC.

Many studies have explored the utility of serologic testing in both the diagnosis of allergy as well as ability to predict tolerance to EH products. For example, specific IgE antibodies to the egg protein ovomucoid have been suggested to be predictors of reactivity to EH egg products, and clinical decision points have been proposed [9]. A recent study suggests a role for the specific IgE/IgG_4_ antibodies to ovalbumin (OVA) and ovomucoid (OVM) in predicting reactivity to extensively heated egg products in egg-allergic children, however this is not a clinically practical or available option [10]. Neither food-specific IgE levels nor SPT responses to commercial food extracts are entirely reliable in identifying children likely to be tolerant of EH milk and egg products [11]. Our proposal to incorporate fresh food SPT in the diagnostic evaluation of milk and egg allergic children may serve as a practical, easy alternative in an allergist’s clinic.

Nevertheless, this study has some limitations. There are inherent flaws in any retrospective study, including lack of blinding of any party. While physician notes from patient encounters were detailed, the data were not originally collected for the purpose of research. Furthermore, the sample size was small, non-homogeneous for one food allergy, and had a much larger egg-allergic cohort. Baseline characteristics between milk and egg allergic individuals were not entirely balanced, and it is unclear whether this is due to a true difference between the two populations. Future studies should assess milk and egg food allergies separately. Although parents were given specific instructions with regards to how to bake the muffins, this factor was not otherwise controlled. Moreover, data were insufficient for calculations of positive predictive value, specificity and sensitivity. Lastly, this study was performed at one centre with the observations and clinical judgment of one clinician; larger multi-clinician, multi-centre trials would be able to better substantiate our findings.

## Conclusions

The majority of patients with milk or egg allergy who had negative SPTs with the EH milk or egg products respectively tolerated the EH form of the food in an OFC. Based on the data collected in this study, skin prick testing with EH food carries a negative predictive value of 94.8% and may be a reliable marker for identifying children likely to tolerate EH milk or egg.

## Abbreviations

SPT: Skin prick test; OFC: Oral food challenge; EH: Extensively heated.

## Competing interests

The authors declare that they have no competing interests.

## Authors’ contributions

ZF helped design the study, conducted the chart review for data collection, analyzed the data and wrote the paper. HK conceived the study and was the primary clinician performing all tests and challenges on subjects. Both authors read and approved the final manuscript.

## Authors’ information

Zein Faraj is a senior medical student at McMaster University, an MD Candidate in the class of 2012. She has previously completed Bachelor of Health Sciences (Honours) degree at McMaster University.

Dr. Harold Kim is an Allergist and Clinical Immunologist practicing in Kitchener, ON. He is an Adjunct Professor at the University of Western Ontario and an Assistant Clinical Professor at McMaster University.
